# Comparison of baseline global gene expression profiles of prostate cancer cell lines LNCaP and DU145

**DOI:** 10.1186/s13104-024-07050-w

**Published:** 2024-12-31

**Authors:** Khalid Ahmed, Zhannur Omarova, Alisalman Sheikh, Gulzhan Abuova, Kulsoom Ghias, Syed Hani Abidi

**Affiliations:** 1 Department of Biological and Biomedical Sciences, Aga Khan University, Karachi, Pakistan; 2 Department of Biomedical Sciences, Nazarbayev University School of Medicine, Astana, Kazakhstan; 3 South Kazakhstan Medical Academy, Shymkent, Kazakhstan

**Keywords:** Prostate cancer, LNCaP, DU145, Cell lines, RNASeq

## Abstract

**Introduction:**

DU145 and LNCaP are classic prostate cancer cell lines. Characterizing their baseline transcriptomics profiles (without any intervention) can offer insights into baseline genetic features and oncogenic pathways that should be considered while interpreting findings after various experimental interventions such as exogenous gene transfection or drug treatment.

**Methods:**

LNCaP and DU145 cell lines were cultured under normal conditions, followed by RNA extraction, cDNA conversion, library preparation, and RNA sequencing using the Illumina NovaSeq platform. The sequences were analyzed to identify differentially expressed genes (DEGs) and for gene ontology (GO) and pathway enrichment.

**Results:**

A total of 3916 and 2301 genes were found to be differentially upregulated and downregulated between LNCaP and DU145 cell lines, respectively. The GO and pathway analysis of up-regulated DEGs indicated significant enrichment of genes involved in extracellular matrix organization and cell-substrate adhesion, while down-regulated genes are involved in epithelial cell migration, cell death regulation, and cell proliferation.

**Conclusion:**

The results showed significant differences in baseline gene expression and cellular pathways that may account for the varying metastatic potentials between LNCaP and DU145 cell lines, which should be considered when interpreting findings after experimental interventions.

**Supplementary Information:**

The online version contains supplementary material available at 10.1186/s13104-024-07050-w.

## Introduction

DU145, a castration-resistant, and LNCaP, a castration-sensitive, cell lines are classic prostate cancer cell lines commonly used in prostate cancer research [1–3]. LNCaP is an androgen-sensitive, low metastatic prostate adenocarcinoma cell line derived from a 50-year-old Caucasian patient with local lymph node metastasis, whereas DU145 is an androgen-insensitive, intermediate metastatic prostate cancer cell line isolated from the brain of a Caucasian prostate cancer patient [4].

DU145 and LNCaP cell lines are often used to study prostate cancer biology, identify therapeutic targets, and perform drug testing involving prostate cancer at the cellular level [5]. DU145 is a model cell line that is used to better understand late-stage prostate cancer and metastasis, study androgen deprivation therapy resistance in prostate cancer, assess the efficacy of anti-tumor drugs and targeted therapy and develop better models of castration-resistant prostate cancer mechanistically at gene and protein level [1]. LNCaP cells are a classic cell line model of androgen-sensitive prostate cancer for understanding androgen receptor cell signaling pathway due to its putative role in the progression of cancer, conducting drug testing in the context of the androgen-sensitive background in prostate cancer cells, and to study genes and proteins that interact with androgen receptor signaling pathways [6].

Characterizing baseline transcriptomics profiles between these cell lines can offer insights into genetic features and oncogenic pathways that may help interpret the findings from various experimental interventions such as exogenous gene transfection, silencing, and drug treatment, among others [7, 8]. For example, due to the lack of full-length ATG5 protein in DU145 cells, autophagy is genetically disrupted; valproic acid treatment cannot induce autophagy in DU145 cells, unlike in LNCaP and PC3 prostate cancer cells [9]. Furthermore, cell lines growing in the laboratory may show phenotypic and genetic drift due to lab culturing adaptation [10, 11].

Results from recent transfection experiments in our laboratory have also shown variability in the expression of certain oncogenes and oncomiRs (data not published), which may have resulted from differences in the baseline expression of these cell lines. Therefore, it is critical to consider this factor when studying mechanisms related to prostate oncogenesis or performing molecular interventions using these cell lines [9]. Analysis of mutations, copy number variations, single nucleotide variants, and fusion genes has been conducted individually in both DU145 and LNCaP cell lines [12–14]. However, to the best of our knowledge, no study has investigated the gene expression profiles and conducted baseline pathway analyses of LNCaP versus DU145 cells.

Here, we report the baseline global gene expression profiles of LNCaP and DU145 cell lines, followed by the gene ontology and pathway analysis. We believe these findings will offer some mechanistic insights related to metastatic potential and other genetic characteristics of DU145 and LNCaP cell lines, which may have implications in oncology research.

## Methods

### Cell Culture

Human prostate cancer cell lines, LNCaP and DU145 (originally purchased from ATCC; cat# CRL-1740 and HTB-81, respectively) were obtained from the stock cultures of the Aga Khan University and cultured/maintained in DMEM medium (Sigma, St. Louis, MO, USA) supplemented with 10% FBS, 1% L-glutamine, and 1% PenStrep at 37˚C in a humidified atmosphere of 5% CO2.

### RNA extraction, library preparation, and RNA sequencing

RNA extraction was performed using the Trizol-chloroform method as described previously [15]. The total RNA concentration was calculated by Quant-IT RiboGreen (Invitrogen, cat#R11490). To assess the integrity of the total RNA, samples are run on the TapeStation RNA ScreenTape (Agilent, cat#5067–5576), and only high-quality RNA preparations with RIN greater than 7.0 were used for RNA library construction. A library was independently prepared with 1 µg of total RNA for each sample by Illumina TruSeq Stranded mRNA Sample Prep Kit (Illumina, Inc., San Diego, CA, USA, cat#20020595). The poly-A-containing mRNA molecules were purified using poly-T‐attached magnetic beads, followed by mRNA fragmentation into small pieces using divalent cations under elevated temperature. The cleaved RNA fragments were copied into first-strand cDNA using SuperScript II reverse transcriptase (Invitrogen, cat#18064014) and random primers and subsequently used for second-strand cDNA synthesis using DNA Polymerase I, RNase H, and dUTP. These cDNA fragments underwent an end-repair process by adding a single adenine (A) base and ligating the adapters. The products were purified and enriched with PCR to create the final cDNA library. The libraries were quantified using KAPA Library Quantification kits for Illumina Sequencing platforms according to the qPCR Quantification Protocol Guide (KAPA BIOSYSTEMS, #KK4854) and qualified using the TapeStation D1000 ScreenTape (Agilent Technologies, # 5067–5582). The indexed libraries were then submitted to an Illumina NovaSeq (Illumina, Inc., San Diego, CA, USA), and the paired-end (2 × 100 bp) sequencing, which was performed by Macrogen Inc., Korea. The experiments were run in duplicate, but cells were pooled at the library preparation step.

### RNAseq analysis & Differential Gene expression (DEGs) analysis

Raw data files in Fastq format were initially quality-checked using FastQC [16], while adapters were trimmed using the FASTP program [17, 18]. The trimmed reads were aligned to the human reference sequence (hg38) using HISAT2 [19]. Subsequently, Cufflinks was used to align reads with the transcripts to compare the differential gene expression across samples [20], while Cuffmerge was used to merge transcript assemblies generated by Cufflinks and remove overlapping transcripts. The differentially expressed genes (DEGs) were identified using Cuffdiff [21]. The DEG data was exported into an MS Excel file, where statistically significant up- and down-regulated DEGs were identified using log2 fold change (log2FC) of > 1 and <-1, respectively, and *p* < 0.05.

We validated our results using the human protein atlas database, where we looked at the gene expression profiles of the top 20 upregulated and top 20 downregulated genes from our dataset in the human protein atlas. The results from the human protein atlas for each of these genes in DU145 and LNCaP cell lines were obtained as the normalized transcription expression values (nTPM) [22]. Since in our dataset, we used LNCaP as the comparator; therefore, we carried out interconversion of dataset values obtained from the human protein atlas using the below-given formula [23, 24].

$$\:Log2\:Fold\:change=\:log2\left(\frac{nTPM\:(Test\:Group-LNCaP)\:}{nTPM\:(Control\:Group-DU145)\:}\right)$$

### Gene ontology (GO) and pathways analyses

The names of the statistically significant DEGs were retrieved using the gProfiler program [25], while GO (cellular, biological, and molecular) and pathway (KEGG, Reactome, and Wiki) enrichment was performed using ShinyGO 0.80 [26]. False Discovery Rate (FDR) cuff-off was kept at a default level of 0.05, and adjusted *p* < 0.05 was used as the cut-off value in all analyses.

## Results and discussion

### Differential Gene expression (DEGs) analysis

**Fig. 1:**
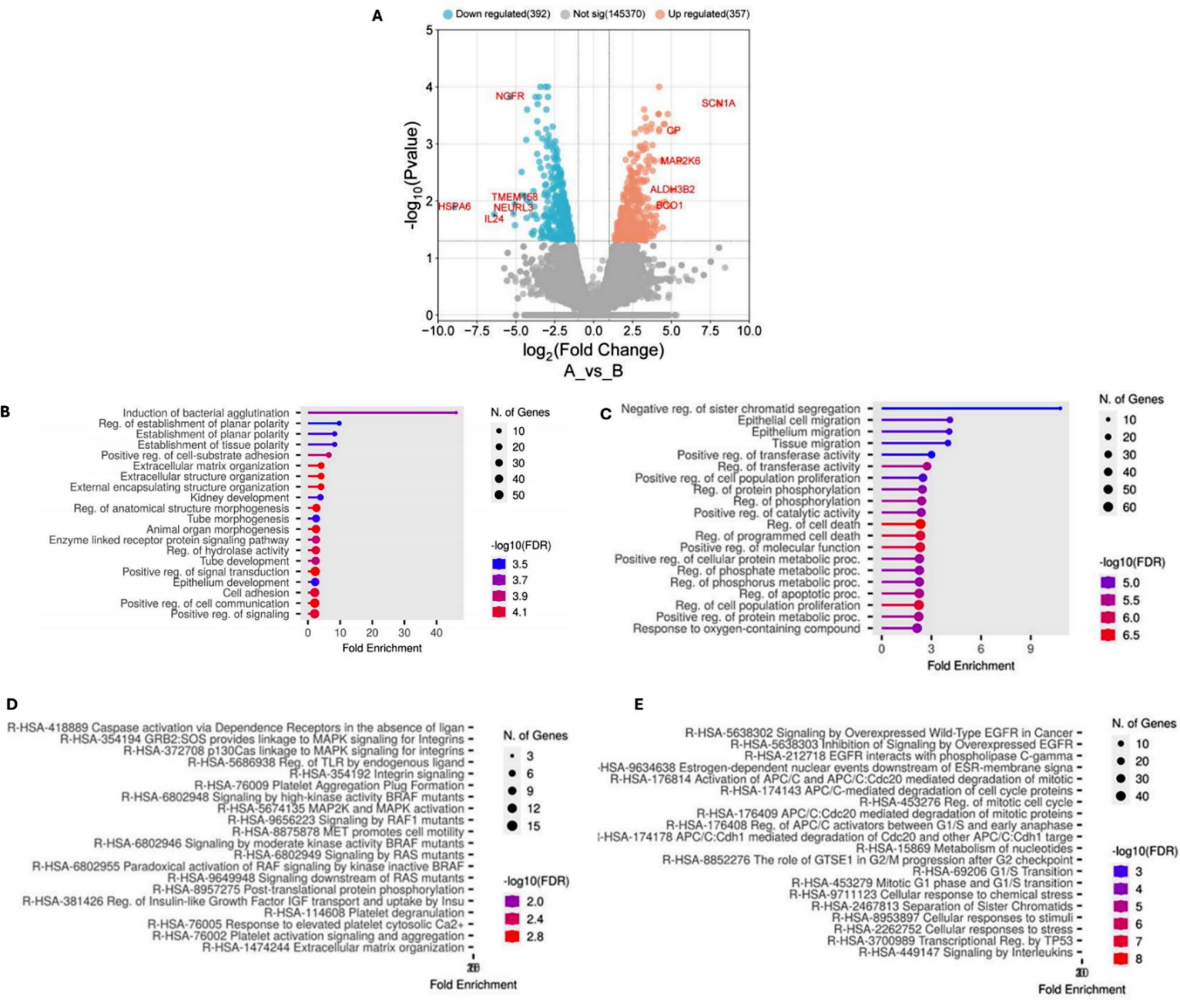
DEG, GO, and pathway enrichment analysis: **A**) Volcano plot showing differentially expressed genes (DEG) in Control DU145 vs. Test LNCaP cells. Enrichment of biological processes (GO) related to **B**) upregulated and **C**) downregulated genes. Curated Reactome analysis of top **D**) upregulated and **E**) down-regulated DEGs

A total of 3916 and 2301 genes were found to be differentially up-regulated and down-regulated between the LNCaP and DU145 cell lines, respectively (Fig. 1A). The top 20 up- and down-regulated genes are described in Table 1. As compared to the DU145 cell line, the top five down-regulated and up-regulated genes in LNCaP cells were *NGFR*,* TMEM158*,* NEURL3*,* IL24*, and *HSPA6; and SCN1A*,* CP*,* MAP2K6*,* ALDH3B2*, and *BCO1* (Fig. 1A; Table 1).

**Table 1:** Top 20 up- and down-regulated genes identified in DEG analysis. The table shows symbols of the genes, log 2FC, and description of top DEGs

Upregulated genes in LNCaP cell lines as compared to DU145 cell lines.			Down-regulated genes in LNCaP cell lines as compared to DU145 cell lines		
Symbols	Log2 FC	Description	Symbols	Log2 FC	Description
*SCN1A*	8.03	sodium voltage-gated channel alpha subunit 1	HSPA6	-8.94	heat shock protein family A (Hsp70) member 6
*MAP2K6*	5.58	mitogen-activated protein kinase kinase 6	*IL24*	-6.39	interleukin 24
*CP*	5.15	ceruloplasmin	*NGFR*	-5.37	nerve growth factor receptor
*ALDH3B2*	5.07	aldehyde dehydrogenase 3 family member B2	*NEURL3*	-5.15	neuralized E3 ubiquitin protein ligase 3
*BCO1*	4.89	beta-carotene oxygenase 1	*TMEM158*	-5.07	transmembrane protein 158
*FGB*	4.76	fibrinogen beta chain	*HMGA2*	-5.05	high mobility group AT-hook 2
*MAML2*	4.58	mastermind like transcriptional coactivator 2	*COX6B2*	-4.64	cytochrome c oxidase subunit 6B2
*FGA*	4.57	fibrinogen alpha chain	*PIK3CG*	-4.63	phosphatidylinositol-4,5-bisphosphate 3-kinase catalytic subunit gamma
*FGG*	4.51	fibrinogen gamma chain	*DUSP15*	-4.45	dual specificity phosphatase 15
*CRLF1*	4.44	cytokine receptor like factor 1	*FOXS1*	-4.45	forkhead box S1
*SFTPB*	4.36	surfactant protein B	*PLPP4*	-4.33	phospholipid phosphatase 4
*B3GNT7*	4.33	UDP-GlcNAc: betaGal beta-1,3-N-acetylglucosaminyltransferase 7	*U2AF1*	-4.31	U2 small nuclear RNA auxiliary factor 1
*FN1*	4.21	fibronectin 1	*TBX2*	-4.30	T-box transcription factor 2
*ST6GALNAC3*	4.21	ST6 N-acetylgalactosaminide alpha-2,6-sialyltransferase 3	*KCNMA1*	-4.28	potassium calcium-activated channel subfamily M alpha 1
*TET1*	4.20	tet methylcytosine dioxygenase 1	*HES7*	-4.10	hes family bHLH transcription factor 7
*FXYD2*	4.18	FXYD domain containing ion transport regulator 2	*HIGD2B*	-3.98	HIG1 hypoxia inducible domain family member 2B
*FXYD6*	4.18	FXYD domain containing ion transport regulator 6	*CHST1*	-3.97	carbohydrate sulfotransferase 1
*FXYD6-FXYD2*	4.18	FXYD6-FXYD2 readthrough	*MIOX*	-3.90	myo-inositol oxygenase
*GPR68*	4.10	G protein-coupled receptor 68	*CCNA1*	-3.86	cyclin A1
*TGFB3*	4.06	transforming growth factor beta 3	*CHST8*	-3.84	carbohydrate sulfotransferase 8

To validate the gene expression profiles of top 20 upregulated and top 20 downregulated genes from our dataset, we compared the expression profiles of those genes in the human protein atlas database. Amongst the downregulated top 20 genes in our dataset, the genes *HSPA6*,* IL24*,* NGFR*,* TMEM158*,* HMGA2*,* COX6B2*,* PIK3CG*,* PLPP4*,* U2AF1*,* TBX2*,* CHST1*, and *CCNA1* were also found to be downregulated in the human protein atlas database (Table S1). Similarly, amongst top 20 upregulated genes in our dataset, the genes *MAP2K6*,* ALDH3B2*, and *FN1* were also found to be upregulated in the human protein atlas database (Table S1). The rest of the genes exhibited the differential gene expression profiles compared to our results.

### Gene ontology and pathways analyses of DEGs

The GO analysis of the up-regulated DEGs in LNCaP cells showed the enrichment of biological processes related to extracellular matrix organization and cell-substrate adhesion (Fig. 1B). The extracellular matrix was the most significantly enriched cellular component and the molecular function (Figures S1A and S1C). Similarly, the Reactome analysis of the up-regulated DEGs revealed enrichment of platelet plug formation, as well as MAPK activation and signaling by RAS, RAF1 and BRAF mutants (Fig. 1D). The KEGG and WikiPathways, however, showed enrichment of pathways related to complement and anticoagulation systems (Figures S1E and S1G).

The GO analysis of the down-regulated DEGs in LNCaP cells as compared to DU145 showed the enrichment of biological processes related to epithelial cell migration, cell death regulation and cell proliferation (Fig. 1C). Key cellular components were associated with the cell cycle, such as the mitotic checkpoint complex, and mitochondrial activity, such as the respiratory chain and cytochrome complexes (Figure S1B). The enriched molecular functions were mainly related to cell-cell adhesion, protein folding and chaperone activity (Figure S1D). Similarly, the Reactome, KEGG and WikiPathways analyses of the downregulated DEGs revealed enrichment of the cell cycle regulation-related pathways (Fig. 1E and Figure S1F and S1H). Additionally, the KEGG and WikiPathways showed enrichment of pathways related to neurological diseases, neuroinflammation and glutamatergic signaling, respectively (Figure S1F and S1H).

## Discussion

This study aimed to examine the baseline differential gene expression between LNCaP and DU145 cell lines to gain insights into the differences between the two commonly used prostate cancer cell line models, which may help in understanding their oncogenic potential and interpret post-intervention (such as, after treatment or transfection) findings.

Previous research has shown LNCaP cells to exhibit comparatively lower invasiveness and metastatic potential as compared to DU145 [27]. These differences may be understood in light of the expression profile and pathways differentially enriched in these cell lines. The pathways associated with extracellular matrix and cell adhesion have important roles in tumor invasion, metastasis, and chemotherapeutic resistance, and these are differentially enriched in these cell lines at baseline [28, 29]. For example, elevated levels of urokinase plasminogen activator and its receptor involved in extracellular matrix degradation and encoded by PLAU and PLAUR are positively correlated with prostate cancer progression and metastasis [30]. Furthermore, another study in LNCaP cells has identified single nucleotide variations associated with extracellular matrix genes [13]. In the analysis reported here, PLAU and PLAUR levels were markedly lower in LNCaP compared to DU145. LNCaP also had significantly lower expression of CXCR4 as compared to DU145; CXCR4 can stimulate cancer cell migration through chemotaxis [31]. HMGA2, which is actively involved in epithelial-mesenchymal transition, migration, and invasion of cancer cells, was also markedly reduced in LNCaP cells as compared to DU145 [32]. Additionally, decreased levels of HMOX1, which encodes heme oxygenase 1, also correlates with a less aggressive type of cancer [33]. This provides mechanistic insight into the less aggressive behavior and more limited metastatic growth of LNCaP cells as compared to DU145 cells.

In addition, LNCaP cells have previously shown a lower proliferation rate as compared to DU145 cells [34]. Our analysis is consistent with this, showing decreased cell cycle progression and cell proliferation-related pathways in LNCaP cells as compared to DU145. However, the enrichment of coagulation and platelet-related pathways by up-regulated DEGs is inconsistent with a previous study, which showed that hormone-insensitive DU145 induced platelet aggregation, while hormone-sensitive LNCaP cells lacked this characteristic [35]. Platelet degranulation pathways were mainly enriched by FN1, FGA, FGB, and FGG, which encode fibronectin 1 and fibrinogen. These proteins are not only components of platelet granules, but also part of the extracellular matrix of cancer cells, and therefore, in this context, are involved in cell adhesion and signal transduction. Therefore, it is important to be cognizant of the background genetic expression profiles in interpreting the intervention results in these prostate cancer cell lines.

We anticipate significant limitations of our study. We used the human protein atlas database to validate the gene expression profiles of the top upregulated and downregulated genes in our dataset [22], however, the standard method for the validation the cell line is the short tandem repeat (STR) analysis [36], which we could not perform due to unavailability of this technique in our setting. Furthermore, due to limited resources we could not outsource this method to other laboratories.

In conclusion, our results showed significant differences in baseline gene expression and cellular pathways that may account for the varying invasion and metastatic potentials between LNCaP and DU145 prostate cancer cell lines. This supports the idea that it is useful to establish the baseline genetic expression profile of a cancer cell line before experimental interventions are undertaken. This may help identify suitable cancer cell models for specific research questions and also in interpreting the findings post-intervention in light of these differences.

## Electronic supplementary material

Below is the link to the electronic supplementary material.

Supplementary Material 1

## Data Availability

All data is available in the manuscript. The RNA sequencing data generated in this study was submitted to GEO Omnibus database and was assigned the accession number GSE283559.
