# Supplementary material for: Comparison of baseline global gene expression profiles of prostate cancer cell lines LNCaP and DU145
Source: BMC Res Notes. 2024 Dec 31;17:398. doi: 10.1186/s13104-024-07050-w (PMC11689513; doi:10.1186/s13104-024-07050-w)
Supplement: Supplementary file 1 — Supplementary Material 1 [file 13104_2024_7050_MOESM1_ESM.docx]

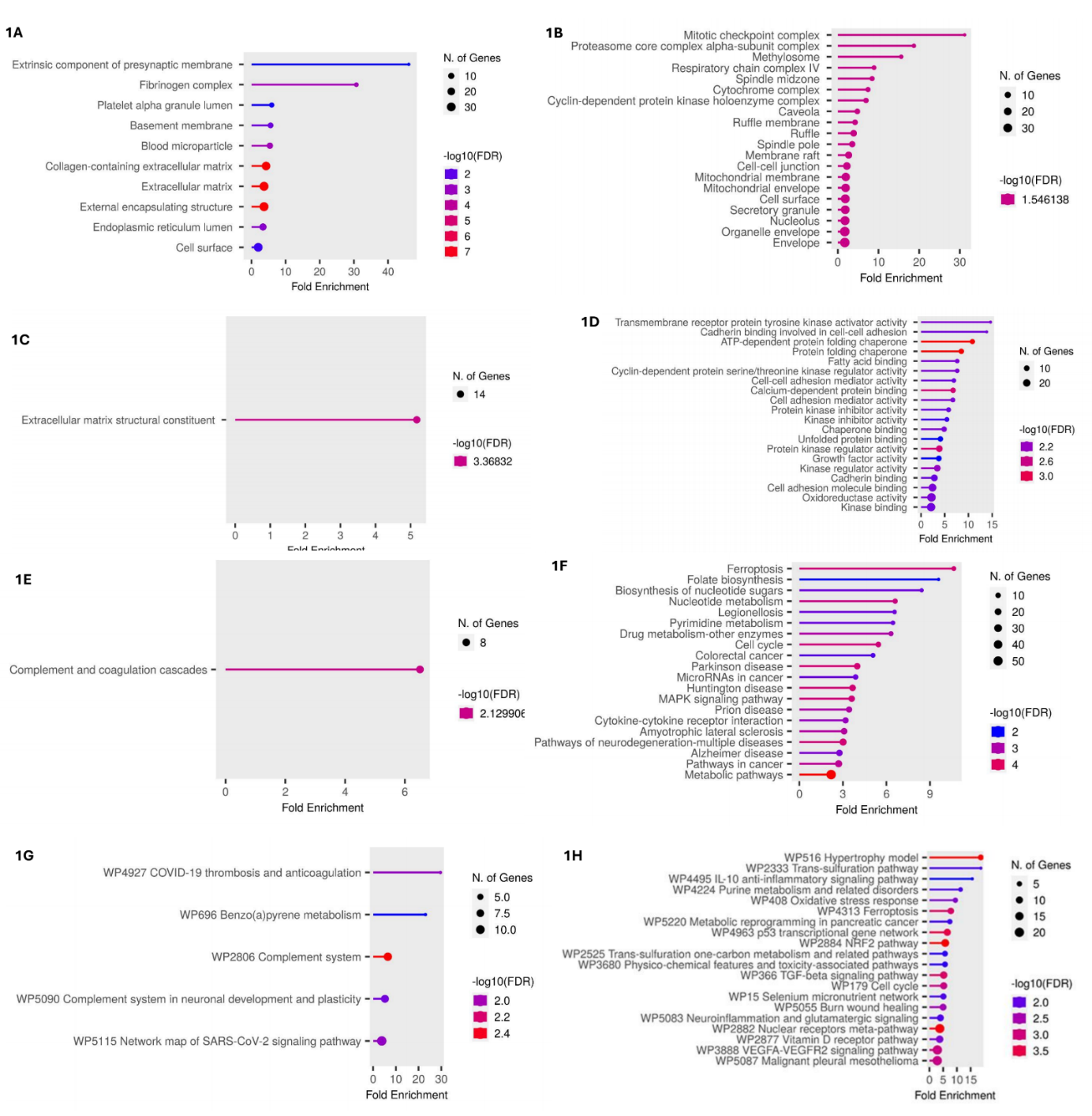


**Figure S1: GO, and pathway enrichment analysis:** Enrichment of cellular components (GO) related to **1A)** upregulated and **1B)** downregulated genes. Enrichment of molecular functions (GO) related to **1C)** upregulated and **1D)** downregulated genes. Enrichment of KEGG pathways related to **1E)** upregulated and **1F)** downregulated genes. Enrichment of Curated WikiPathways pathways related to **1G)** upregulated and **1H)** downregulated genes.

**Table S1. The human protein atlas-based validation of the gene expression profiles of top upregulated and downregulated genes in our dataset.** The table shows symbols of the genes, log 2FC of our RNAseq results versus log2FC as per human protein atlas database. The values that are highlighted in bold indicate the agreement in between the gene expression profiles in our dataset and the human protein atlas.

| **Symbols** | **Log2 FC as per RNAseq results** | **Log2 FC as per Human Protein Atlas** | **Symbols** | **Log2 FC as per RNAseq results** | **Log2 FC as per Human Protein Atlas** |
| --- | --- | --- | --- | --- | --- |
| *SCN1A* | 8.03 | 0 | *HSPA6* | **-8.94** | **-0.68** |
| *MAP2K6* | **5.58** | **2.59** | *IL24* | **-6.39** | **-21** |
| *CP* | 5.15 | 0 | *NGFR* | **-5.37** | **-18.19** |
| *ALDH3B2* | **5.07** | **24.83** | *NEURL3* | -5.15 | 1 |
| *BCO1* | 4.89 | -18.93 | *TMEM158* | **-5.07** | **-2.48** |
| *FGB* | 4.76 | -18.93 | *HMGA2* | **-5.05** | **-4.7** |
| *MAML2* | 4.58 | 0 | *COX6B2* | **-4.64** | **-5.04** |
| *FGA* | 4.57 | -18.61 | *PIK3CG* | **-4.63** | **-16.61** |
| *FGG* | 4.51 | -18.61 | *DUSP15* | -4.45 | 0.58 |
| *CRLF1* | 4.44 | -4 | *FOXS1* | -4.45 | 0 |
| *SFTPB* | 4.36 | -0.81 | *PLPP4* | **-4.33** | **-27.27** |
| *B3GNT7* | 4.33 | -0.48 | *U2AF1* | **-4.31** | **-0.81** |
| *FN1* | **4.21** | **2.19** | *TBX2* | **-4.30** | **-19.35** |
| *ST6GALNAC3* | 4.21 | -18.93 | *KCNMA1* | -4.28 | 0.25 |
| *TET1* | 4.20 | 0.32 | *HES7* | -4.10 | -1.89 |
| *FXYD2* | 4.18 | -18.61 | *HIGD2B* | -3.98 | 0 |
| *FXYD6* | 4.18 | -6.28 | *CHST1* | **-3.97** | **-16.61** |
| *FXYD6-FXYD2* | 4.18 | 0 | *MIOX* | -3.90 | 19.6 |
| *GPR68* | 4.10 | -0.32 | *CCNA1* | **-3.86** | **-22.33** |
| *TGFB3* | 4.06 | -2.81 | *CHST8* | -3.84 | 0 |
